# Coproduction of Low-Barrier Hepatitis C Virus and HIV Care for People Who Use Drugs in a Rural Community: Brief Qualitative Report

**DOI:** 10.2196/47395

**Published:** 2023-09-20

**Authors:** Shoshana H Bardach, Amanda N Perry, Elizabeth Eccles, Elizabeth A Carpenter-Song, Ryan Fowler, Erin M Miers, Anais Ovalle, David de Gijsel

**Affiliations:** 1 The Dartmouth Institute for Health Policy and Clinical Practice Geisel School of Medicine at Dartmouth College Lebanon, NH United States; 2 Section of Infectious Diseases & International Health Dartmouth-Hitchcock Medical Center Lebanon, NH United States; 3 Department of Anthropology Dartmouth College Hanover, NH United States; 4 HIV/HCV Resource Center Lebanon, NH United States; 5 Park Nicollet Health Services Minneapolis, MN United States; 6 Care New England Warwick, RI United States; 7 Better Life Partners Manchester, NH United States

**Keywords:** hepatitis C, HIV, coproducing care, testing, people who use drugs, HCV, rural community

## Abstract

**Background:**

People who inject drugs are experiencing syndemic conditions with increasing risk of infection with hepatitis C (HCV) and HIV. However, rates of accessing HCV and HIV testing and treatment among people who inject drugs are low for various reasons, including the criminalization of drug use, which leads to a focus on treating drug use rather than caring for drug users. For many people who inject drugs, health care becomes a form of structural violence, resulting in traumatic experiences, fear of police violence, unmet needs, and avoidance of medical care. There is a clear need for novel approaches to health care delivery for people who inject drugs.

**Objective:**

This study aimed to analyze the process of a multidisciplinary team—encompassing health care professionals, community representatives, researchers, and people with lived experience using drugs—that was formed to develop a deep understanding of the experiences of people who inject drugs and local ecosystem opportunities and constraints to inform the cocreation of low-barrier, innovative HCV or HIV care in a rural community. Given the need for innovative approaches to redesigning health care, we sought to identify challenges and tensions encountered in this process and strategies for overcoming these challenges.

**Methods:**

Analysis was based on an in-depth review of meeting notes from the project year, followed by member-checking with the project team to revise and expand upon the challenges encountered and strategies identified to navigate these challenges.

**Results:**

Challenges and tensions included: scoping the project, setting the pace and urgency of the work, adapting to web-based work, navigating ethics and practice of payment, defining success, and situating the project for sustainability. Strategies to navigate these challenges included: dedicated effort to building personal and meaningful connections, fostering mutual respect, identifying common ground to make shared decisions, and redefining successes.

**Conclusions:**

While cocreated care presents challenges, the resulting program is strengthened by challenging assumptions and carefully considering various perspectives to think creatively and productively about solutions.

## Introduction

### Hepatitis C Virus and HIV Among People Who Inject Drugs—Treatment Challenges

Infections such as hepatitis C (HCV) and HIV disproportionally affect people who inject drugs. Globally, over half of people who inject drugs are infected with HCV, and nearly 1 in 5 with HIV [1-5]. Many people who inject drugs are unaware of their HCV or HIV status, and rates of accessing treatment are low. For instance, fewer than 10% of those infected with HCV receive treatment [6], increasing morbidity and contributing to ongoing virus spread [7].

Several factors contribute to the low rates at which people who inject drugs access treatment. People who inject drugs are often stereotyped and treated poorly in professional settings; many have histories of traumatizing experiences with medical care [8,9]. These experiences cause health care mistrust and fear of being criminalized for drug use rather than treated for health needs [10]. Differences between the values and goals of clinicians and people who inject drugs can exacerbate difficulties between groups [11]. Medical professionals’ focus on achieving abstinence from drug use leads to missed opportunities for people who inject drugs to engage in health care if people who inject drugs are not interested or able to stop using drugs. Treatment for HCV is not always made available to people with active drug use. Some service providers exclude people who inject drugs from HCV treatment due to concerns about poor treatment adherence and risk for reinfection, despite national guidelines explicitly recommending the inclusion of people who inject drugs in HCV treatment [12,13]. Even in places without treatment restrictions, myths and knowledge gaps may limit treatment access [14-17]. Further, even when individuals are aware that HCV is curable, people who inject drugs may not prioritize treatment for an infection that is asymptomatic and not immediately life-threatening over more pressing needs associated with chaotic drug use. HCV treatment may also be less appealing for those who witnessed individuals taking first-generation HCV medications that had profound side effects [18].

In rural areas, compounded barriers affect treatment access, including long distances to care and less health care availability, lack of transportation, limited internet and phone connectivity, lower socioeconomic status and associated concerns regarding medication costs, and unstable housing [19-21]. These same factors may also help explain why people who inject drugs in rural areas receive HCV or HIV testing less frequently than their urban counterparts [22]. Barriers to treatment in rural areas coexist with increasing opioid use and opioid-related mortality rates [23].

### The Value of Coproducing Care

Extensive research highlights that coproducing care and programs increases the likelihood that the resulting programs match the needs of the target communities, resulting in better health outcomes, patient satisfaction, and even cost savings [24-26]. Principles of community engagement highlight the importance of fostering relationships, building off existing trust, being flexible and responsive, promoting ongoing engagement through mutual understanding, user-centeredness, and reciprocity, empowering community members, being willing to be questioned and challenged, creating a safe and supportive environment, and respecting differences [27]. Following these aforementioned principles and providing opportunities for individuals with lived experience of drug use to have longitudinal roles within the project helps to avoid tokenism and promote genuine engagement [28].

### Innovating HCV or HIV Care Delivery

With 1 year of funding from a health care delivery innovation laboratory [29], we used a human-centered design approach to develop a program to facilitate the connection between health care providers and populations at risk for sex- and drug-related harms [30,31]. We assembled a multidisciplinary team of health care professionals, community representatives, researchers, and people who inject drugs. We invited health care professionals with a wide range of expertise in infectious diseases and addiction: an infectious diseases and addiction medicine physician, an HIV nurse care manager, an HIV outreach nurse, and a psychologist embedded in the HIV program with a focus on trauma and addiction. To augment clinical perspectives, the team included a medical anthropologist with experience working with rural communities as well as several harm reduction specialists with lived experience of drug use who work for community-based organizations serving people who struggle with addiction, those are, a syringe service program and an addiction treatment program. The team also included a person with lived experience of drug use as a patient innovation partner; the patient innovation partner role was designed by the health care delivery innovation laboratory to ensure teams included an embedded team member who was encouraged to share the patient perspective throughout the design journey. In composing the team, we aimed to include multiple perspectives from within as well as from outside the health care system. Due to COVID-19 and social distancing recommendations, project team meetings occurred digitally via Zoom (Zoom Video Communications).

To be responsive to the needs and preferences of people who inject drugs and to ensure a range of perspectives and experiences with drug use and health care informed the program design, the team developed a community advisory board (CAB). Most CAB members were current or former clients of the syringe service program, and one was an employee of another syringe service program. All 8 CAB members had a history of injection drug use and 4 were currently using. While not all CAB members shared their HCV or HIV status, 3 indicated prior treatment for HCV and one indicated being actively treated for HIV. The CAB included 4 men and 4 women and, consistent with area demographics, was mostly White (7 White and 1 Black). CAB members lived all over the rural northeast, including New Hampshire, Vermont, Maine, and upstate New York.

The CAB met with members of the project team via the web, once or twice per month for 6 months, providing feedback and generating ideas regarding educational and promotional materials, testing and referral processes, and outreach strategies. CAB members were also given opportunities to participate in individual interviews to share additional program development ideas and suggestions.

A notable contribution of the CAB was the development of the name for the program; they came up with “To the Point,” which resulted from a conversation around sticking to the goals of the program and a play on words when considering the point of a needle transmitting virus. “To the Point” was preferred to the project’s working name, “Connect to Cure,” as curing HCV or HIV is often not of immediate interest—especially when infection status is not yet known—among people who inject drugs.

This work resulted in a new care pathway grounded in principles of trauma-informed care and harm reduction that is embedded within an existing syringe service program. The program developed is a novel community-based, peer-led, HCV or HIV testing service in rural Vermont and New Hampshire. The program is responsive to the schedules and preferred testing locations of people who inject drugs and relies on staff already embedded within the community with lived experience of drug use and established trust. During testing encounters, clients are offered immediate, digital connections to medical providers and are given harm reduction information and supplies including naloxone, safe injection equipment, wound care supplies, and information about HCV and HIV.

While the project team was deeply committed to designing a program responsive to community preferences and needs, the process of coproduction was fraught with difficulties. The purpose of this paper is to shed light on the challenges this multidisciplinary team encountered along the way in coproducing this new care model and the strategies used to navigate these challenges.

## Methods

### Overview

A general inductive approach was used to analyze the data, whereby instances of challenge or tension were used to identify broader categories of challenge, without any preconceived notions or theories guiding the analysis [32]. While throughout the project period team members had informally identified various challenges and tensions throughout the process, at the conclusion of the 12 months of the project, the first author carefully reviewed all notes from the project year. Notes included weekly meeting notes from throughout the project, project-related emails, and monthly CAB meeting notes. For all notes, excerpts that reflected differing perspectives, tensions, or uncertainty regarding how best to proceed with program design were pulled. These instances were then sorted into thematic areas of tension or challenge, with thematic categories refined iteratively, as additional data were reviewed. The resulting challenge areas were brought back to the full project team on multiple occurrences—both in writing and via verbal discussion—as a form of member-checking to review for accuracy, thoroughness, and appropriate categorization. Because the analysis focused on challenges encountered by the project’s multidisciplinary team throughout the project year, CAB members were not part of this member-checking process.

### Ethical Considerations

The team’s work was reviewed by Dartmouth Health’s institutional review board and determined to be quality improvement, not human participants’ research. All project team members were compensated for their time on project-related activities, including this analysis, either through effort brought out through their institution or through payment for time, depending on their work situation. CAB members were sent a US $25 gift card for their participation in each meeting. In addition, clients were incentivized with US $25 gift cards for participating in the HCV or HIV testing and subsequent steps in the care cascade (ie, follow-up blood work, a clinical visit, initiating medications, finishing medication, and final blood work).

## Results

In total, 6 areas of challenge in coproducing care were identified: scoping the project, pace and urgency, adapting to web-based work, navigating the ethics and process of fair payment, defining success, and situating for sustainability. Each of these is discussed below.

### Scoping the Project

When this project launched, the focus—originally defined by the project lead, an infectious disease physician—was on helping individuals who already knew they were HCV-infected to follow through on receiving treatment and cure. However, through the design thinking process, it became clear that this focus was too downstream and was missing the larger need to help people in the community learn their infection status, and many were hesitant to engage with health care. Accordingly, the project’s focus shifted to increasing HCV or HIV testing and linkages to health care. Social injustices and fundamental public health problems were identified as the project progressed. While the team recognized that testing for HCV or HIV was limited, other more immediate medical needs such as wound care, management of acute infections, and mental health treatment were identified as unaddressed. Further, basic needs for food and shelter were unmet, and access to phone service, stable internet, and transportation remained challenging. Several team members wanted to tackle these broader health and social challenges—and tension emerged between staying focused on project goals and keeping these issues front of mind. Academia encourages specific aims that are both measurable and achievable; scope creep and losing focus can compromise traditional notions of success or measurable progress. Accordingly, the team maintained the HCV or HIV testing focus, acknowledging that the team’s composition and time constraints were not aligned for addressing these broader public health issues, but integrated an awareness of social determinants of health and health inequalities into the program design. We managed these tensions by framing the initial work as a proof-of-concept pilot, being explicit about the program’s current limits, but maintaining the goal of future expansion to address greater, more immediate needs.

### Pace and Urgency

Health care quality improvement is often slow, with various processes and approvals required for changes [33], but developing procedures to ensure safety and confidentiality is critical. Moving slowly to ensure adequate data are collected to evaluate program effectiveness to support publications and grants enables broader impact potential but typically does not have immediate benefit. For those immersed in the community faced with people suffering daily, there is an urgent need for change and frustration with the slow, bureaucratic processes to make health care changes. The low tolerance for risk and error in health care is at odds with the substantial and urgent health needs that are not being addressed for people who inject drugs. Accordingly, there was tension between the clinical and research members of the team’s desire to pause to develop strategies to ensure safety, confidentiality, and rigorous data collection and the community representatives’ and people who inject drugs’ desire to bring testing into the community and offer support as soon as possible. This tension over urgency is also evident in writing about the work; peer-reviewed academic publications tend to encourage a neutral voice, but a neutral tone may mask the emotion involved and the urgent need for change.

### Adapting to Web-Based Work

The project team’s work occurred almost exclusively via the web. Interacting remotely makes it challenging to develop the personal connections that bond teams and build trust and belonging [34]. Special efforts to connect were necessary, such as: dedicating time to empathize with personal struggles and celebrate successes, creating opportunities to engage in occasional, in-person meet-ups, and recognizing the passion that team members brought to the work. These explicit efforts to strengthen relationships and build trust are essential to successful coproduction [35]. In addition, the team actively attempted to reduce hierarchies in team structure and minimize power differentials, striving to build consensus by seeking out and listening to all team members, and fostering mutual respect in the search for common ground. In the absence of common ground, individuals may abandon the work, reinforcing existing cultural divides. Accordingly, the search for common ground requires an ongoing, continued, and explicit effort.

### Navigating Ethics and Practice of Fair Payment

The team had to navigate the ethics of fairly compensating people who inject drugs for sharing their experiences to inform program design—both through the CAB and initial design thinking interviews—while maintaining a noncoercive relationship and abiding by prohibitive institutional rules regarding hiring and payment. Prior research suggests that compensation can build trust and demonstrate reciprocity and respect, enabling engagement to evolve [36]. While there may be concern about people who inject drugs redirecting cash toward drug use, research does not validate this concern [37] and questions the ethics of “item restrictions” in financial support programs (eg, food assistance). Institutional rules prevented employing CAB members in the health system due to a required drug test. Other compensation options relied on quarterly payments—a long wait for individuals with minimal financial resources. These limitations can prevent people who inject drugs from engaging in such projects. Navigating these payment challenges also raised questions about the role and ethical responsibility of large, well-funded institutions to their surrounding communities. Ultimately, the team chose to use cash gift cards to balance regulation with autonomy and respect for privacy.

### Redefining Success

After the initial month of testing, no one who tested positive for HCV had connected to health care, despite efforts to provide low-barrier engagement opportunities. Several team members saw this as a failure; others highlighted that more people knowing their viral status was itself a success and empowering individuals to take the next step in the care cascade, when and if they decide to, was essential. Recognizing the pervasive distrust of the medical community, the team redefined notions of success. Accordingly, a positive, nonjudgmental, clinical experience may hold value in and of itself [38]. The team viewed these as corrective emotional experiences; when individuals engaged in medical-like encounters without the stigma they have previously experienced, future health care receptivity may increase. The team also acknowledged that individuals may move through this care cascade at different speeds—and adjusted expectations around the immediacy of follow-up. The project’s time constraints encouraged rapid measurement, yet people who inject drugs may take months to take the next step from testing to treatment. By month 2 of the program, the program began to see some connections to health care.

### Situating for Sustainability

The goal of the health care innovation laboratory is for new care pathways to be sustainable after the initial year of funding. Sustainability is vital so that gains in community trust or headway in overcoming barriers to health care are preserved. Return on investment calculations can justify new positions for initiatives exclusively situated within the health care system. This project, however, does not immediately translate into resource savings or new revenue generation for the health care institution. While providing compassionate linkages from the community into the health care setting may introduce new patients into the system or encourage earlier care for infections and wounds rather than costly emergency visits, potential revenue and cost savings are delayed. Accordingly, the team was challenged with advocating for and obtaining ongoing financial support from the health care system while operating the program outside of the traditional walls of the institution.

## Discussion

This coproduction process revealed several insights that may help others planning to engage in similar partnerships. Committed partnerships enable teams to move the needle with respect to care; however, even with dedication, creating change remains difficult. Building bridges to overcome cultural barriers, by engaging people who inject drugs in the team and program design and including team members with experience both as people who inject drugs and in service delivery, helped develop a shared understanding of challenges, for example, people who inject drugs’ experience of health care trauma and mistrust. By acknowledging deep-seated challenges, and validating and acknowledging the values of people who inject drugs, the resulting program design was sensitive to community needs and health care limitations.

The importance of ongoing open conversation and support is not to be understated. There may be a frankness or directness that emerges that is uncomfortable, but this honesty should be recognized and appreciated as an opportunity to address personal frustrations and strengthen relationships [39,40]. Refocusing on a shared mission, that defies traditional ideas around working with people who inject drugs that views stopping substance use as a primary success indicator, and acknowledging and celebrating small successes, helps build connection and is at least a step toward re-energizing and countering the burnout frequently experienced by individuals working in this area [41]. These partnerships benefit from individuals entering the work with mental flexibility and a willingness to challenge assumptions and think creatively and productively about solutions; the design thinking approach used for this program’s development encouraged such innovative thinking [42].

This work also highlights the important role that community partnerships can play in sustainability, leveraging each group’s strengths to support common goals and shared missions to help the communities they serve. This work is currently being sustained through a combination of health center staff investment, syringe service program staff support, and philanthropic funding. These partnerships were possible due to strategic and comprehensive communication with health care and community organization leaders, creating shared buy-in, support, and ownership for the project’s ongoing success [43]. By distributing the work and costs of running the program, and by clearly communicating the need for change, program goals, and the work needed to achieve those goals, resistance to change is reduced [44]. These collaborations will likely evolve over time, as incentives, constraints, and priorities shift. Efforts to evaluate program impact on clients, providers, and the communities in which this work occurs, will hopefully facilitate ongoing investment from all parties [45,46]. The ongoing work also requires shared responsibility and flexibility on the part of the health system, to incorporate care innovations that disrupt usual practices. Fostering a willingness to work through ambiguity can help establish an institutional context that can accommodate change.

There are several limitations to acknowledge in this analysis. First, since meetings were not audio-recorded, challenges identified in the coproduction process may have been overlooked if they were not reflected in the team meeting notes or actively recalled by team members. Consequently, it is possible that additional challenges were encountered that are not reflected in the discussion above. Further, because the notes were taken by a member of the innovation laboratory staff, it is possible the academic lens through which notes were taken may have led to some challenges being overlooked. However, the diversity of the team, including an anthropologist sensitive to team dynamics, and the iterative discussions of findings hopefully minimized potential omissions. In addition, while this analysis focuses on the coproduction process among the project team members, future research could also examine challenges in the context of the CAB. Despite these limitations, this analysis demonstrates that several tensions occur throughout the coproduction process. The negotiations and thoughtful considerations of various perspectives that emerged from these tensions supported the development of a program that is sensitive to the preferences and needs of the population it seeks to serve. Future work will evaluate the initial outcomes of this program.

## Abbreviations

CAB: community advisory board
HCV: hepatitis C virus
